# Quantum-Chemical Prediction of Molecular and Electronic Structure of Carbon-Nitrogen Chemical Compound with Unusual Ratio Atoms: C(N_20_)

**DOI:** 10.3390/ijms24065172

**Published:** 2023-03-08

**Authors:** Oleg V. Mikhailov, Denis V. Chachkov

**Affiliations:** 1 Department of Analytical Chemistry, Certification and Quality Management, Kazan National Research Technological University, K. Marx Street 68, 420015 Kazan, Russia; 2 Kazan Department of Joint Supercomputer Center of Russian Academy of Sciences—Branch of Federal Scientific Center “Scientific Research Institute for System Analysis of the RAS”, Lobachevskii Street 2/31, 420111 Kazan, Russia; de2005c@gmail.com

**Keywords:** carbon-nitrogen compound, C(N_20_), molecular structure, quantum-chemical calculation method

## Abstract

Using various versions of quantum-chemical calculation, namely four versions of density functional theory (DFT), (DFT B3PW91/TZVP, DFT M06/TZVP, DFT B3PW91/Def2TZVP, and DFT M06/Def2TZVP) and two versions of the MP method (MP2/TZVP and MP3/TZVP), the existence possibility of the carbon-nitrogen-containing compound having an unusual M: nitrogen ratio of 1:20, unknown for these elements at present, was shown. Structural parameters data are presented; it was noted that, as may be expected, CN_4_ grouping has practically a tetrahedral structure, and the chemical bond lengths formed by nitrogen atoms and a carbon atom in the frameworks of each of the calculation methods indicated above are equal to each other. Thermodynamical parameters, NBO analysis data, and HOMO/LUMO images for this compound are also presented. A good agreement between the calculated data obtained using the above three quantum-chemical methods was noticed, too.

## 1. Introduction

As is well known, carbon and nitrogen together form a very significant number of chemical compounds—neutral as well as cations and anions. At present, as a minimum, several dozen neutral compounds are known to contain atoms of only these two elements; the simplest (and most known) of these are the cyanogens C_2_N_2_ (cyanogen N≡C–C≡N, isocyanogen C=N–C≡N, and diisocyanogen C=N–N=C) and tricarbon tetranitride or, simply carbon nitride C_3_N_4_, first described more than 100 years ago and considered in more detail in a number of works, in particular [1,2,3,4,5,6,7,8,9,10,11,12,13,14,15]. The carbon:nitrogen ratio in known (C,N)-binuclear compounds containing atoms of only those two elements varies from 69:1 in diheterofullerene (C_69_N)_2_ [16] to 1:12 in tetraazidomethane C(N_3_)_4_ [17] and (dodecaazadodecatetraene-1,4, 7,10)carbon(IV) C(N_12_) [18] with structural formulas **I** and **II**, respectively.

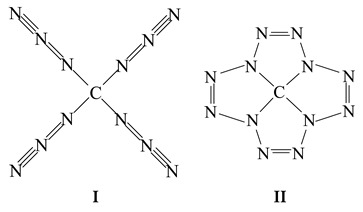



The existence of compound **I** was confirmed experimentally [17], while the existence of compound **II** was predicted based on the results of quantum chemical calculations carried out using DFT B3PW91/TZVP, MP2/TZVP, and MP3/TZVP methods [18]. Accordingly, these two C(N12) isomers are the most nitrogen-rich compounds, containing only C and N atoms. However, the specified ratio C:N = 1:12 is not the maximum possible, and one can in principle assume the possibility of the existence of carbon-nitrogen-containing chemical compounds with an even greater number of nitrogen atoms per carbon atom. An example of such a compound is tetra(pentaazolato)carbon(IV) C(N_5_)_4_, having structural formula **III** in which the C:N ratio is equal to 1:20.

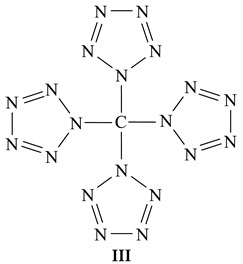



Such binuclear compounds, containing only nitrogen and carbon atoms, where the C:N ratio is less than 1:10, are of considerable interest as potential high-energetic substances [17,19,20]. As far as is known, compound **III** has not yet been considered in the literature even theoretically; in this connection, the given communication is devoted to the consideration of the question of the possibility of its existence and, in the case of a positive conclusion, to the determination of the parameters of its molecular structure and thermodynamic characteristics using modern quantum-chemical methods of calculation, namely the various versions of density functional theory (DFT) and Möller–Plesset perturbation theory (MP).

## 2. Results and Discussion

According to the data of each of the above six methods of quantum chemical calculation, the chemical compound of the composition with the gross formula C(N_20_) is capable of independent existence as an isolated molecule, at least in the gas phase. The lengths of chemical bonds and bond angles between atoms in this chemical compound, calculated by these methods, are presented in Table 1. As it is easy to see when comparing the data presented in it, the values of the above key parameters of the molecular structure are very close to each other, and therefore it seems appropriate to discuss them together. 

Taking into account the fact that the carbon atom in the C(N_20_) compound under consideration with the above structural formula III is bonded to four nitrogen atoms, we can expect that these N atoms should be located at the vertices of a regular tetrahedron or a polyhedron close to it. Indeed, the calculation of the molecular structure of C(N_20_) using each of the six quantum chemical methods we used indicates that a grouping of four nitrogen atoms bonded to a carbon atom forms an almost regular tetrahedron. Wherein, firstly, the C–N bond lengths are equal to each other (although they somewhat depend on the calculation method); secondly, the angles (NCN) differ little from each other, and their values are close to the values of similar angles in a regular tetrahedron (109.5°) (Table 1). In this regard, it is worth noting that the non-bonding angles (NNN) formed by those nitrogen atoms that are bonded to the C atom are close to 60° in each of the six methods (i.e., to the angles of a regular triangle). The pentazole fragments are strictly coplanar since the sum of the internal bond angles in each of them is the same and coincides with the sum of the internal angles in a flat pentagon (540°).

**Figure 1:**
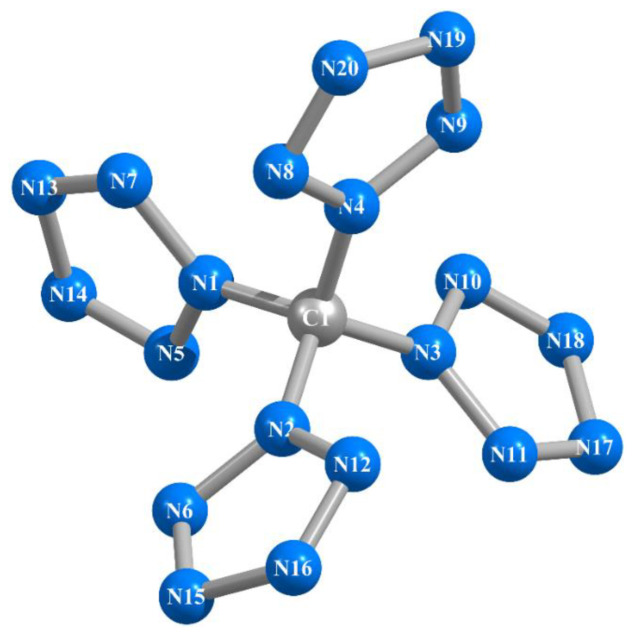
Molecular structure of the C(N_20_) compound obtained as a result of MP3/TZVP quantum-chemical calculation.

As should be expected, within the framework of each of these methods, they are completely identical to each other, although the sets of these angles differ somewhat from each other; the lengths of the N–N chemical bonds in them are equal in pairs (Table 1). That is characteristic, the C–N bond lines are in the same plane as the planes of the corresponding pentazole fragments. In general, in qualitative terms (in appearance), the molecular structures of this compound obtained by these six calculation methods show almost complete similarity with each other; an example of an image of such a structure is shown in Figure 1. The values of the electrical dipole moments of this compound obtained by each of the DFT B3PW91/TZVP, MP2/TZVP, and MP3/TZVP methods practically do not differ from 0.00 Debye units, which, taking into account the quasi-tetrahedral molecular structure of C(N_20_) (point group of symmetry *D_2_*) obtained by each of these methods, seems to be quite expected.

The key data of the NBO analysis, namely the values of the effective charges on the carbon and nitrogen atoms in the test compound, obtained by the DFT, MP2, and MP3 methods, are presented in Table 2. The selected data of the NBO analysis of the C(N_20_) compound under examination are presented in the Supplementary Materials. As can be seen from these data, the effective charges on individual atoms calculated by the above methods with an accuracy of 0.01 are quite close to each other. Be that as it may, these values, judging by their absolute values, are much less than +4.00 ē (for the C1 atom) and –1.00 ē (for the N1–N4 atoms bonded to the C1 atom by chemical bonds), which would be the case if all chemical bonds between C and N atoms were ionic. In our opinion, this fact indicates the presence of a high degree of electron density delocalization in this compound. The ground state of this compound in the framework of each of these methods is a spin singlet (*M_S_* = 1), and the values of the operator of the square of the intrinsic angular momentum of the total spin of the system <S**2> are equal to 0. In addition, according to the calculation data in the framework of any of the above six methods, the nearest excited state with a different MS value, namely, the spin triplet (*M_S_* = 3), is significantly higher in energy than the ground state (for example, according to the B3PW91/TZVP method, by 403 kJ/mol). Testing the wave functions of the ground state for stability using the standard procedure STABLE = OPT showed that the wave function of the ground state at *M_S_* = 1 is stable with respect to the perturbations under consideration.

**Figure 2:**
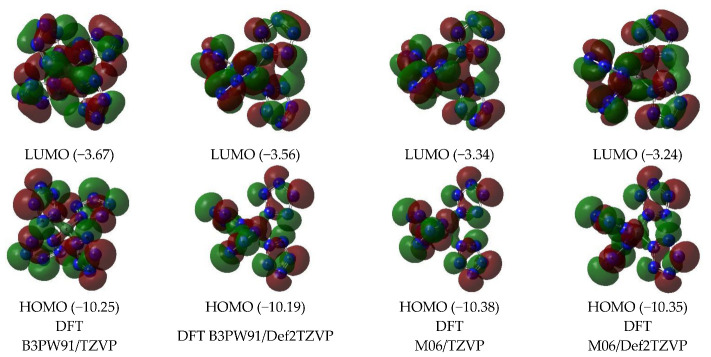
The images of highest occupied (HOMO) and lowest unoccupied (LUMO) molecular orbitals in the C(N_20_) (ground state—spin singlet, *M_S_* = 1) according to DFT B3PW91/TZVP, DFT B3PW91/Def2TZVP, DFT M06/TZVP, and DFT M06/Def2TZVP methods. The energies values of these molecular orbitals (in brackets) are given in eV.

Images of the highest occupied and lowest vacant (unoccupied) molecular orbitals (HOMO and LUMO, respectively) obtained by using DFT B3PW91/TZVP, DFT B3PW91/Def2TZVP, DFT M06/TZVP, and DFT M06/Def2TZVP quantum-chemical methods are presented in Figure 2. As can be seen from this, the LUMO shapes obtained by each of these three methods are quite close to each other. As for HOMO, the above similarity is observed only for orbitals obtained by DFT methods.

B3PW91/Def2TZVP, M06/TZVP, and M06/Def2TZVP, while HOMO obtained by the B3PW91/TZVP method has a different shape (Figure 2). The energies of these HOMO and LUMO obtained by DFT methods, as can be seen from Figure 2, are quite close to each other. However, they differ significantly from the energies of similar MO obtained using MP methods, which, taking into account the above, also seem quite natural and predictable.

The standard thermodynamic parameters of formation (∆_f_*H*^0^, *S*^0^, and ∆_f_*G*^0^) for the chemical compound under study are given in Table 3. As may be seen from it, all these parameters are positive, and, therefore, this compound, as should be expected, cannot be obtained from the most thermodynamically stable simple substances formed by carbon and nitrogen (i.e., graphite and molecular nitrogen N_2_). In connection with the foregoing, it is of particular interest to consider the reaction of the interaction of C(N_20_) with molecular oxygen, which proceeds in the gas phase according to Reaction (1)

C(N_20_) (gas) + O_2_ (gas) → CO_2_ (gas) + 10N_2_ (gas) (1)

and the decomposition reactions of this compound according to any of the Reactions (2)–(4)

C(N_20_) (gas) → C (gas) + 10N_2_ (gas) (2)

C(N_20_) (gas) → C (graphite) + 10N_2_ (gas) (3)

C(N_20_) (gas) → C (diamond) + 10N_2_ (gas) (4)

The thermodynamic parameters of all these reactions are presented in Table 4. As can be seen from the data indicated in it, the reaction of the interaction of the compound C(N_20_) under consideration with molecular oxygen (1) is strongly exothermic, since its enthalpy ∆_r_*H*^0^ < 0, and the numerical value of this thermodynamic parameter according to its absolute value is very high (more than 2000 kJ). However, since the value of its entropy ∆_r_*S*^0^ > 0, then according to the well-known Gibbs–Helmholtz Equation (5),

∆_r_*G*(T) = ∆_r_*H*^0^ − T∆_r_*S*^0^ (5)

at any temperature T, the relation ∆_r_*G*(T) < 0 will take place. Therefore, the process described by Equation (1) is irreversible. In this regard, it is interesting to note that a similar situation occurs for Reactions (2)–(4) (Table 4), the last of which, in principle, can be used even to obtain an allotropic modification of carbon—diamond within the isobaric process.

## 3. Method

To carry out the quantum-chemical calculation, in this work, we used the density functional theory (DFT), which combines the standard extended split valence basis set TZVP and the hybrid functional B3PW91, described in detail in Refs [22,23,24] and used by us, in particular, in [25,26,27]. The use of the B3PW91/TZVP method, in this case, is because, according to [22,23,24], it allows one to obtain, as a rule, the most accurate (i.e., close to experimental) values of the geometric parameters of molecular structures, as well as much more accurate values of thermodynamic and other physical-chemical parameters in comparison with other variants of the DFT method. For comparison, other, later versions of the DFT method were also used, namely the M06 functional [28] and the newer redefinitions of the TZVP basis set—Def2TZVP [29]. The calculations were carried out using the Gaussian09 program package [30]. As in our previous articles, in which this method of calculation was used [25,26,27], the correspondence of the found stationary points to the energy minima in all cases was proved by calculating the second derivatives of the energy to the coordinates of the atoms; wherein, all equilibrium structures corresponding to the minimum points on the potential energy surfaces had only real (and, moreover, always positive) frequency values. Of the optimized structures for further consideration, the one with the lowest total energy was selected. Since the structure of C(N_20_) may be a priori nontrivial, in order not to encounter a probable overestimation of the stability of such a structure when calculating by DFT methods, we decided to use an “honest” ab initio method for calculation along with DFT methods, as far as our capabilities allow. As it is well known, the DFT methods when calculating structures with a nontrivial distribution of electron density can, in some cases, lead to an incorrect order of orbitals and even an incorrect molecular structure. To exclude such a possibility, we decided to carry out the calculation using some ab initio quantum chemical methods. However, unfortunately, it was not possible to complete the calculation with such methods with a stricter account of electronic correlation as CCSD and QCISD due to high computational costs. That is why, in addition to the calculation by the DFT B3PW91/TZVP, DFT B3PW91/Def2TZVP, DFT M06/TZVP, and DFT M06/Def2TZVP methods, as an alternative, we used Möller-Plesset perturbation theory methods [31]. Unfortunately, we could only use the methods of second and third-order perturbation theory, namely MP2 [32] and MP3 [33], in combination with the extended split valence TZVP basis set, each of which is noticeably less computationally intensive than the CCSD and QCISD methods. (At the moment, it was also not possible to complete the calculation using the MP4 method, since in terms of computational complexity it is comparable to the CCSD method). We could not use other ab initio methods for the calculation since the technical and time resources at our disposal did not allow us to do this.

Natural bond orbital (NBO) analysis was carried out, using NBO version 3.1, integrated with the Gaussian09 program package [30], according to the methodology described in detail [34]. NBO methods are well known for their excellent numerical stability and convergence with respect to basis set expansion that is sensibly proportionate to the convergence of energy and other calculated wavefunction properties (unlike Mulliken analysis and related overlap-dependent methods in this case). The standard thermodynamic parameters of formation (∆_f_*H*^0^, *S*^0^, and ∆_f_*G*^0^) for the C(N_20_) compound under examination were calculated using the G4 method described in [35].

## 4. Conclusions

As follows from the above, the presented results of quantum chemical calculations performed using six different methods, namely four DFT methods (B3PW91/TZVP, B3PW91/Def2TZVP, M06/TZVP, and M06/Def2TZVP) and two MP methods (MP2/TZVP, MP3/TZVP), unambiguously testify in favor of the possibility of the existence of a new (and not yet experimentally discovered) chemical compound of carbon and nitrogen with the composition C(N_20_) [C(N_5_)_4_]. This substance is characterized by an unusual (and so far the highest among all currently known binary compounds of carbon and nitrogen) ratio between the number of N atoms and the number of C atoms (20:1) and has a quasi-tetrahedral molecular structure. At the same time, the use of DFT methods with B3PW91 and M06 potentials both with the TZVP basis and with the more advanced Def2TZVP basis leads to practically the same results (although the Def2TZVP set contains a much larger number of basis functions—651 versus 399—and its use requires much more energy—time costs). A similar situation occurs when comparing the results of the calculations of C(N_20_) by the above variants of the DFT method with the results of the calculations by the MP2 and MP3 methods, the use of which is even more expensive. Thus, there is every reason to state that the DFT methods generally correctly describe the molecular structure of the compound under study, at least in a qualitative sense.

Judging by the very high value of ∆_f_*G*^0^ (>2500 kJ/mol), the C(N_20_) [C(N_5_)_4_] compound under consideration is indeed a high-energy substance; if it is obtained experimentally, it will undoubtedly find some practical application, at least in the above capacity. Predicting the possibility of the existence of such an exotic chemical and modeling its molecular and electronic structures using modern quantum chemical calculations can serve as a very useful tool in solving the problems associated with this synthesis. On the other hand, its synthesis may be of great importance for the further development of physical chemistry and chemical technology of both its constituent chemical elements, which, as is known, play an extremely important role in nature.

## Figures and Tables

**Table 1:** Bond lengths and bond angles in the C(N_20_) [C(N_5_)_4_] calculated by and various versions of DFT and MP methods.

Structural Parameter	Calculated by					
	DFT B3PW91/ TZVP	DFT B3PW91/ Def2TZVP	DFT M06/ TZVP	DFT M06/ Def2TZVP	MP2/ TZVP	MP3/ TZVP
Bond lengths						
C–N bond lengths, pm						
C1N1	144.9	145.0	144.4	144.5	144.1	144.4
C1N2	144.9	145.0	144.4	144.5	144.1	144.4
C1N3	144.9	145.0	144.4	144.5	144.1	144.4
C1N4	144.9	145.0	144.4	144.5	144.1	144.4
N–N bond lengths, pm						
N1N5	133.1	132.7	133.1	132.7	133.4	132.8
N5N14	127.5	127.5	127.0	127.0	131.2	127.5
N14N13	136.0	136.5	136.5	136.2	135.3	136.5
N13N7	127.5	127.5	127.0	127.0	131.2	127.6
N7N1	133.1	132.7	133.1	132.7	133.5	132.8
N2N6	133.1	132.7	133.1	132.7	133.5	132.8
N6N15	127.5	127.5	127.0	127.0	131.2	127.5
N15N16	136.0	136.5	136.5	136.2	135.3	136.5
N16N12	127.5	127.5	127.0	127.0	131.2	127.6
N12N2	133.1	132.7	133.1	132.7	133.4	132.8
N3N10	133.1	132.7	133.1	132.7	133.4	132.8
N10N18	127.5	127.5	127.0	127.0	131.2	127.5
N18N17	136.0	136.5	136.5	136.2	135.3	136.5
N17N11	127.5	127.5	127.0	127.0	131.2	127.6
N11N3	133.1	132.7	133.1	132.7	133.5	132.8
N4N8	133.1	132.7	133.1	132.7	133.4	132.8
N8N20	127.5	127.5	127.0	127.0	131.2	127.5
N20N19	136.0	136.5	136.5	136.2	135.3	136.5
N19N9	127.5	127.5	127.0	127.0	131.2	127.6
N9N4	133.1	132.7	133.1	132.7	133.5	132.8
**Bond angles**						
Bond angles in the 5-numbered ring (N1N5N14N13N7), deg						
N1N5N14	104.6	104.7	104.7	104.8	103.4	104.5
N5N14N13	109.4	109.3	109.3	109.2	109.6	109.3
N14N13N7	109.4	109.4	109.4	109.3	109.8	109.3
N13N7N1	104.5	104.6	104.6	104.8	103.3	104.5
N7N1N5	112.1	112.0	112.0	111.9	113.9	112.4
Bond angles sum (BAS^1^), deg	540.0	540.0	540.0	540.0	540.0	540.0
Bond angles in the 5-numbered ring (N2N6N15N16N12), deg						
N2N6N15	104.5	104.6	104.6	104.8	103.3	104.5
N6N15N16	109.5	109.4	109.4	109.3	109.8	109.3
N15N16N12	109.4	109.3	109.3	109.2	109.6	109.3
N16N12N2	104.5	104.7	104.7	104.8	103.4	104.5
N12N2N6	112.1	112.0	112.0	111.9	113.9	112.4
Bond angles sum (BAS^2^), deg	540.0	540.0	540.0	540.0	540.0	540.0
Bond angles in the 5-numbered ring (N3N10N18N17N11), deg						
N3N10N18	104.6	104.7	104.7	104.8	103.4	104.5
N10N18N17	109.4	109.3	109.3	109.2	109.6	109.3
N18N17N11	109.4	109.4	109.4	109.3	109.8	109.3
N17N11N3	104.5	104.6	104.6	104.8	103.3	104.5
N11N3N10	112.1	112.0	112.0	111.9	113.9	112.4
Bond angles sum (BAS^3^), deg	540.0	540.0	540.0	540.0	540.0	540.0
Bond angles in the 5-numbered ring (N4N8N20N19N9), deg						
N4N8N20	104.6	104.7	104.7	104.8	103.4	104.5
N8N20N19	109.4	109.3	109.3	109.2	109.6	109.3
N20N19N9	109.4	109.4	109.4	109.3	109.8	109.3
N19N9N4	104.5	104.6	104.6	104.8	103.3	104.5
N9N4N8	112.1	112.0	112.0	111.9	113.9	112.4
Bond angles sum (BAS^4^), deg	540.0	540.0	540.0	540.0	540.0	540.0
(NCN) bond angles between carbon and two nitrogen atoms, deg						
N1C1N2	110.1	110.2	110.0	110.1	110.1	108.1
N1C1N3	110.1	110.2	110.0	110.1	110.1	108.1
N1C1N4	110.1	110.2	110.0	110.1	110.1	110.2
N2C1N3	110.1	110.2	110.0	110.1	110.1	110.2
N2C1N4	108.2	108.0	108.3	108.2	108.2	108.1
N3C1N4	108.2	108.0	108.3	108.2	108.2	110.2

**Table 2:** NBO analysis data for the C(N20) calculated by DFT B3PW91/TZVP, DFT B3PW91/Def2TZVP, DFT M06/TZVP, DFT M06/Def2TZVP, MP2/TZVP, and MP3/TZVP methods.

Calculation Method	Effective Charge on Atom, in Elementary Charge Units					
	C1	N1, N2, N3, N4	N5, N8, N10, N12	N6, N7, N9, N11	N13, N15, N17, N19	N14, N16, N18, N20
B3PW91/TZVP	+0.55	−0.06	−0.00	−0.01	−0.03	−0.03
B3PW91/Def2TZVP	+0.56	−0.06	−0.00	−0.01	−0.03	−0.03
M06/TZVP	+0.57	−0.07	+0.00	−0.00	−0.03	−0.03
M06/Def2TZVP	+0.59	−0.07	+0.00	−0.01	−0.03	−0.03
MP2/TZVP	+0.68	−0.12	+0.02	+0.02	−0.04	−0.04
MP3/TZVP	+0.68	−0.13	+0.02	+0.02	−0.04	−0.04

**Table 3:** Standard thermodynamic parameters of formation at T = 298.15 K and P = 101,325 Pa (∆_f_*H*^0^, *S*^0^ and ∆_f_*G*^0^) for C(N_20_) calculated by B3PW91/TZVP and G4 methods.

Calculation Method	∆_f_*H*^0^, kJ/mol	*S*^0^, J/mol∙K	∆_f_*G*^0^, kJ/mol
B3PW91/TZVP	2213.5	551.5	2616.0
G4	2052.1	559.8	2452.2

**Table 4:** Standard enthalpies (∆_r_*H*^0^) and standard entropies (∆_r_*S*^0^) of the Reactions (1)–(4) at T = 298.15 K and P = 101,325 Pa according to data of B3PW91/TZVP and G4 methods.

Calculation Method	Reaction							
	(1)		(2)		(3) *		(4) *	
	∆_r_*H*^0^, kJ	∆_r_*S*^0^, J∙K^−1^	∆_r_*H*^0^, kJ	∆_r_*S*^0^, J∙K^−1^	∆_r_*H*^0^, kJ	∆_r_*S*^0^, J∙K^−1^	∆_r_*H*^0^, kJ	∆_r_*S*^0^, J∙K^−1^
B3PW91/ TZVP	−2288.3	1347.1	−1196.4	1520.2	−1913.2	1368.0	−1911.4	1364.6
G4	−2459.0	1378.5	−1344.5	1513.0	−2061.2	1360.8	−2059.4	1357.4

* When calculating ∆_r_*H*^0^ and ∆_r_*S*^0^ in the case of Reactions (3) and (4) for C (graphite) and C (diamond), the experimental values of the standard thermodynamic parameters ∆_f_*H*^0^ and *S*^0^ of these carbon modifications were taken [21].

## Data Availability

No unpublished data was created or analyzed in this article.

## Supplementary Materials

The following supporting information can be downloaded at: https://www.mdpi.com/article/10.3390/ijms24065172/s1.
