# Supplementary material for: Quantum-Chemical Prediction of Molecular and Electronic Structure of Carbon-Nitrogen Chemical Compound with Unusual Ratio Atoms: C(N20)
Source: Int J Mol Sci. 2023 Mar 8;24(6):5172. doi: 10.3390/ijms24065172 (PMC10049734; doi:10.3390/ijms24065172)
Supplement: Supplementary file 1 [file ijms-24-05172-s001.zip › ijms-2257142-supplementary.pdf]

**B3PW91/TZVP**

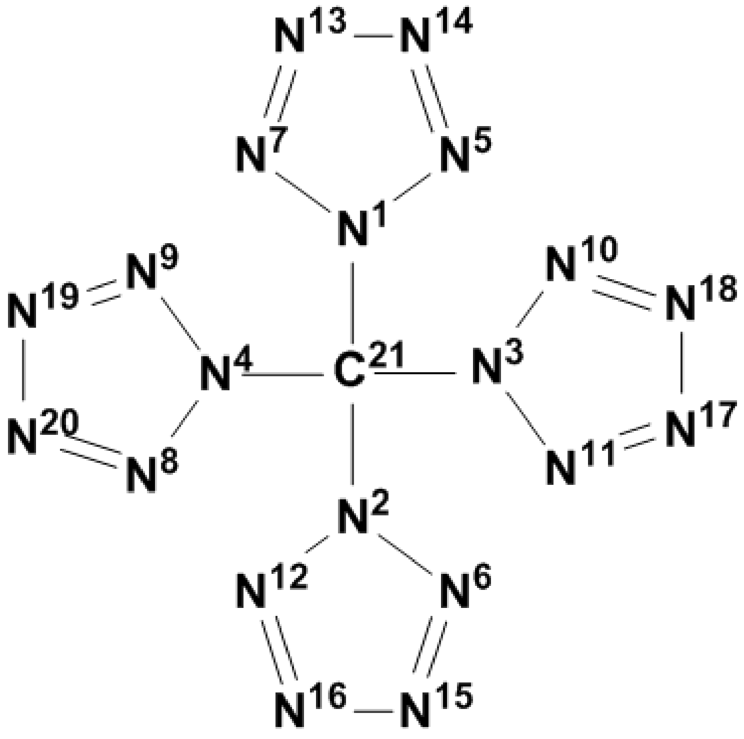


Dipole moment (Debye) = 0.0001

```
Mulliken charges:
     1  N   -0.037528
     2  N   -0.037526
     3  N   -0.037537
     4  N   -0.037536
     5  N    0.081107
     6  N    0.026486
     7  N    0.026486
     8  N    0.081113
     9  N    0.026483
    10  N    0.081127
    11  N    0.026479
    12  N    0.081111
    13  N   -0.033234
    14  N   -0.034172
    15  N   -0.033236
    16  N   -0.034176
    17  N   -0.033235
    18  N   -0.034176
    19  N   -0.033242
    20  N   -0.034165
    21  C   -0.010628
Sum of Mulliken charges =  -0.00000
```

**ΔE(multipl.=1) = 0.0 кДж/моль**

**Alpha occupied eigenvalues (highest) = -10.252728 eV**
**Alpha virtual eigenvalues (lowest) = -3.667908 eV**

<S**2> = 0.0000

Summary of Natural Population Analysis:

| Atom | No | Natural Charge | Natural Population Core | Valence | Rydberg | Total |
|---|---|---|---|---|---|---|
| N | 1 | -0.06191 | 1.99952 | 5.03440 | 0.02799 | 7.06191 |
| N | 2 | -0.06191 | 1.99952 | 5.03440 | 0.02799 | 7.06191 |
| N | 3 | -0.06192 | 1.99952 | 5.03441 | 0.02799 | 7.06192 |
| N | 4 | -0.06191 | 1.99952 | 5.03440 | 0.02799 | 7.06191 |
| N | 5 | -0.00069 | 1.99958 | 4.97325 | 0.02786 | 7.00069 |
| N | 6 | -0.00818 | 1.99959 | 4.98045 | 0.02815 | 7.00818 |
| N | 7 | -0.00818 | 1.99959 | 4.98045 | 0.02815 | 7.00818 |
| N | 8 | -0.00069 | 1.99958 | 4.97325 | 0.02786 | 7.00069 |
| N | 9 | -0.00818 | 1.99959 | 4.98045 | 0.02815 | 7.00818 |
| N | 10 | -0.00069 | 1.99958 | 4.97325 | 0.02786 | 7.00069 |
| N | 11 | -0.00818 | 1.99959 | 4.98044 | 0.02815 | 7.00818 |
| N | 12 | -0.00069 | 1.99958 | 4.97325 | 0.02786 | 7.00069 |
| N | 13 | -0.03466 | 1.99970 | 5.00537 | 0.02959 | 7.03466 |
| N | 14 | -0.03258 | 1.99970 | 5.00322 | 0.02965 | 7.03258 |
| N | 15 | -0.03466 | 1.99970 | 5.00537 | 0.02959 | 7.03466 |
| N | 16 | -0.03258 | 1.99970 | 5.00322 | 0.02965 | 7.03258 |
| N | 17 | -0.03466 | 1.99970 | 5.00537 | 0.02959 | 7.03466 |
| N | 18 | -0.03257 | 1.99970 | 5.00322 | 0.02965 | 7.03257 |
| N | 19 | -0.03466 | 1.99970 | 5.00537 | 0.02959 | 7.03466 |
| N | 20 | -0.03258 | 1.99970 | 5.00322 | 0.02965 | 7.03258 |
| C | 21 | 0.55209 | 1.99908 | 3.40324 | 0.04560 | 5.44791 |
| * Total * | | -0.00000 | 41.99144 | 103.39001 | 0.61854 | 146.00000 |

**Optimized Parameters**
**(Angstroms and Degrees)**

| **Bond lenghts** | | **Torsion (dihedral) angles** | |
|---|---|---|---|
| R(1,5) | 1.3306 | D(7,1,5,14) | 0.1547 |
| R(1,7) | 1.3309 | D(21,1,5,14) | 178.9049 |
| R(1,21) | 1.4485 | D(5,1,7,13) | -0.1516 |
| R(2,6) | 1.3309 | D(21,1,7,13) | -178.8819 |
| R(2,12) | 1.3306 | D(5,1,21,2) | -75.48 |
| R(2,21) | 1.4485 | D(5,1,21,3) | 44.9099 |
| R(3,10) | 1.3306 | D(5,1,21,4) | 165.2992 |
| R(3,11) | 1.3309 | D(7,1,21,2) | 103.1126 |
| R(3,21) | 1.4485 | D(7,1,21,3) | -136.4975 |
| R(4,8) | 1.3306 | D(7,1,21,4) | -16.1082 |
| R(4,9) | 1.3309 | D(12,2,6,15) | 0.1461 |
| R(4,21) | 1.4485 | D(21,2,6,15) | 178.8769 |
| R(5,14) | 1.2747 | D(6,2,12,16) | -0.1473 |
| R(6,15) | 1.275 | D(21,2,12,16) | -178.898 |
| R(7,13) | 1.275 | D(6,2,21,1) | 16.1088 |
| R(8,20) | 1.2747 | D(6,2,21,3) | -103.1126 |
| R(9,19) | 1.275 | D(6,2,21,4) | 136.4983 |
| R(10,18) | 1.2747 | D(12,2,21,1) | -165.298 |
| R(11,17) | 1.275 | D(12,2,21,3) | 75.4805 |
| R(12,16) | 1.2747 | D(12,2,21,4) | -44.9086 |
| R(13,14) | 1.3599 | D(11,3,10,18) | 0.1404 |
| R(15,16) | 1.3599 | D(21,3,10,18) | 178.8919 |
| R(17,18) | 1.3599 | D(10,3,11,17) | -0.1546 |
| R(19,20) | 1.3599 | D(21,3,11,17) | -178.8862 |
| **Bond angles** | | D(10,3,21,1) | 44.9136 |
| A(5,1,7) | 112.0793 | D(10,3,21,2) | 165.3036 |
| A(5,1,21) | 123.2839 | D(10,3,21,4) | -75.4759 |
| A(7,1,21) | 124.624 | D(11,3,21,1) | -136.4924 |
| A(6,2,12) | 112.0794 | D(11,3,21,2) | -16.1024 |
| A(6,2,21) | 124.6233 | D(11,3,21,4) | 103.1181 |
| A(12,2,21) | 123.2845 | D(9,4,8,20) | -0.1513 |
| A(10,3,11) | 112.0789 | D(21,4,8,20) | -178.903 |
| A(10,3,21) | 123.2844 | D(8,4,9,19) | 0.1549 |
| A(11,3,21) | 124.6239 | D(21,4,9,19) | 178.8867 |
| A(8,4,9) | 112.0793 | D(8,4,21,1) | 75.4751 |
| A(8,4,21) | 123.2841 | D(8,4,21,2) | -44.9145 |
| A(9,4,21) | 124.6238 | D(8,4,21,3) | -165.3037 |
| A(1,5,14) | 104.5869 | D(9,4,21,1) | -103.1191 |
| A(2,6,15) | 104.5024 | D(9,4,21,2) | 136.4913 |
| A(1,7,13) | 104.5028 | D(9,4,21,3) | 16.102 |
| A(4,8,20) | 104.5874 | D(1,5,14,13) | -0.0945 |
| A(4,9,19) | 104.5023 | D(2,6,15,16) | -0.0854 |
| A(3,10,18) | 104.5875 | D(1,7,13,14) | 0.0869 |
| A(3,11,17) | 104.5021 | D(4,8,20,19) | 0.0861 |
| A(2,12,16) | 104.5872 | D(4,9,19,20) | -0.0953 |
| A(7,13,14) | 109.464 | D(3,10,18,17) | -0.0695 |
| A(5,14,13) | 109.3668 | D(3,11,17,18) | 0.1053 |
| A(6,15,16) | 109.4647 | D(2,12,16,15) | 0.0884 |
| A(12,16,15) | 109.3661 | D(7,13,14,5) | 0.005 |
| A(11,17,18) | 109.4657 | D(6,15,16,12) | -0.0019 |
| A(10,18,17) | 109.3656 | D(11,17,18,10) | -0.0237 |
| A(9,19,20) | 109.4651 | D(9,19,20,8) | 0.0061 |

| | | |
|---|---|---|
| A(8,20,19) | 109.3656 | |
| A(1,21,2) | 110.1164 | |
| A(1,21,3) | 108.1892 | |
| A(1,21,4) | 110.1161 | |
| A(2,21,3) | 110.1162 | |
| A(2,21,4) | 108.1887 | |
| A(3,21,4) | 110.1159 | |

Cartesian coordinates (standard orientation)

| Center Number | Atomic Number | Atomic Type | Coordinates (Angstroms) X | Y | Z |
|---|---|---|---|---|---|
| 1 | 7 | 0 | 0.855207 | 0.686172 | -0.946602 |
| 2 | 7 | 0 | -0.844433 | -0.952628 | -0.691119 |
| 3 | 7 | 0 | 0.843730 | -0.685082 | 0.957571 |
| 4 | 7 | 0 | -0.854502 | 0.951581 | 0.680099 |
| 5 | 7 | 0 | 1.925521 | 0.125239 | -1.503659 |
| 6 | 7 | 0 | -0.636139 | -1.387032 | -1.931748 |
| 7 | 7 | 0 | 0.657272 | 1.928002 | -1.382420 |
| 8 | 7 | 0 | -1.917484 | 1.514958 | 0.111573 |
| 9 | 7 | 0 | -0.662912 | 1.386141 | 1.923358 |
| 10 | 7 | 0 | 1.906001 | -0.122789 | 1.528508 |
| 11 | 7 | 0 | 0.641782 | -1.927199 | 1.390725 |
| 12 | 7 | 0 | -1.914040 | -1.517260 | -0.136443 |
| 13 | 7 | 0 | 1.608678 | 2.132745 | -2.206200 |
| 14 | 7 | 0 | 2.390171 | 1.022284 | -2.281009 |
| 15 | 7 | 0 | -1.580399 | -2.217623 | -2.141977 |
| 16 | 7 | 0 | -2.367817 | -2.298004 | -1.036103 |
| 17 | 7 | 0 | 1.582728 | -2.130757 | 2.226720 |
| 18 | 7 | 0 | 2.361524 | -1.019167 | 2.312005 |
| 19 | 7 | 0 | -1.610951 | 2.215427 | 2.121386 |
| 20 | 7 | 0 | -2.383941 | 2.295018 | 1.005321 |
| 21 | 6 | 0 | 0.000005 | 0.000009 | -0.000014 |

**B3PW91/Def2TZVP**

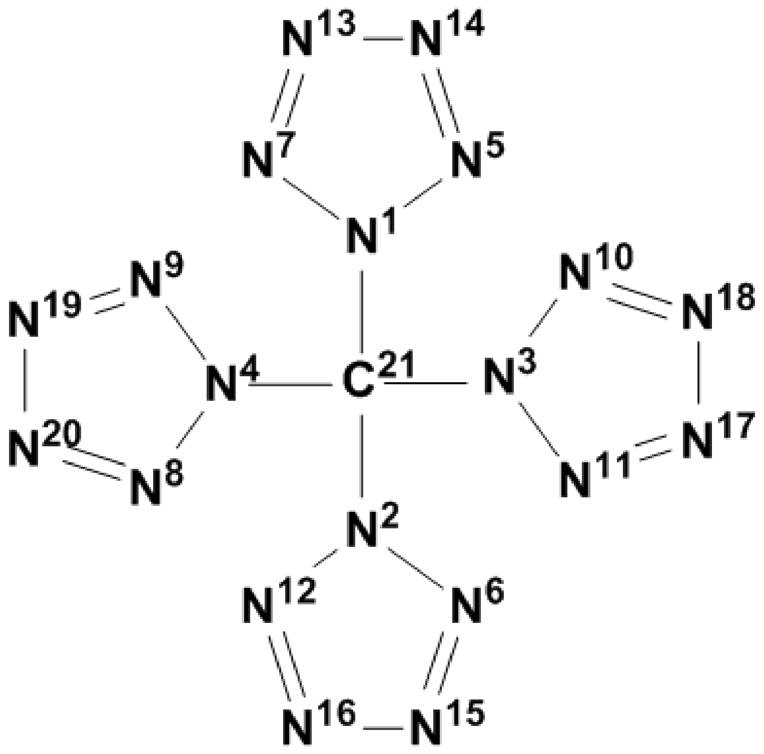


Dipole moment (Debye) = 0.0000

Mulliken charges:

| | | |
|---|---|---|
| 1 | N | 0.186575 |
| 2 | N | 0.186576 |
| 3 | N | 0.186575 |
| 4 | N | 0.186575 |
| 5 | N | -0.046765 |
| 6 | N | -0.074417 |
| 7 | N | -0.074418 |
| 8 | N | -0.046766 |
| 9 | N | -0.074415 |
| 10 | N | -0.046762 |
| 11 | N | -0.074421 |
| 12 | N | -0.046767 |
| 13 | N | -0.063438 |
| 14 | N | -0.068674 |
| 15 | N | -0.063441 |
| 16 | N | -0.068671 |
| 17 | N | -0.063438 |
| 18 | N | -0.068674 |
| 19 | N | -0.063442 |
| 20 | N | -0.068671 |
| 21 | C | 0.266879 |

Sum of Mulliken charges = -0.00000

**ΔE(multipl.=1) = 0.0 кДж/моль**

**Alpha occupied eigenvalues (highest) = -10.1917776 eV**
**Alpha virtual eigenvalues (lowest) = -3.5555307 eV**

<S**2> = 0.0000

Summary of Natural Population Analysis:

| Atom | No | Natural Charge | Natural Population Core | Valence | Rydberg | Total |
|---|---|---|---|---|---|---|
| N | 1 | -0.05751 | 1.99950 | 5.02630 | 0.03172 | 7.05751 |
| N | 2 | -0.05751 | 1.99950 | 5.02630 | 0.03172 | 7.05751 |
| N | 3 | -0.05751 | 1.99950 | 5.02630 | 0.03172 | 7.05751 |
| N | 4 | -0.05751 | 1.99950 | 5.02630 | 0.03172 | 7.05751 |
| N | 5 | -0.00186 | 1.99957 | 4.96818 | 0.03411 | 7.00186 |
| N | 6 | -0.01038 | 1.99957 | 4.97648 | 0.03433 | 7.01038 |
| N | 7 | -0.01038 | 1.99957 | 4.97648 | 0.03433 | 7.01038 |
| N | 8 | -0.00186 | 1.99957 | 4.96818 | 0.03411 | 7.00186 |
| N | 9 | -0.01038 | 1.99957 | 4.97648 | 0.03433 | 7.01038 |
| N | 10 | -0.00186 | 1.99957 | 4.96818 | 0.03411 | 7.00186 |
| N | 11 | -0.01038 | 1.99957 | 4.97648 | 0.03433 | 7.01038 |
| N | 12 | -0.00186 | 1.99957 | 4.96818 | 0.03411 | 7.00186 |
| N | 13 | -0.03672 | 1.99971 | 5.00070 | 0.03630 | 7.03672 |
| N | 14 | -0.03460 | 1.99971 | 4.99828 | 0.03661 | 7.03460 |
| N | 15 | -0.03672 | 1.99971 | 5.00070 | 0.03630 | 7.03672 |
| N | 16 | -0.03460 | 1.99971 | 4.99828 | 0.03661 | 7.03460 |
| N | 17 | -0.03672 | 1.99971 | 5.00071 | 0.03630 | 7.03672 |
| N | 18 | -0.03459 | 1.99971 | 4.99828 | 0.03661 | 7.03459 |
| N | 19 | -0.03672 | 1.99971 | 5.00071 | 0.03630 | 7.03672 |
| N | 20 | -0.03460 | 1.99971 | 4.99828 | 0.03661 | 7.03460 |
| C | 21 | 0.56424 | 1.99907 | 3.39887 | 0.03782 | 5.43576 |
| * Total * | | 0.00000 | 41.99133 | 103.27861 | 0.73006 | 146.00000 |

**Optimized Parameters**
**(Angstroms and Degrees)**

| Bond lengths | | Torsion (dihedral) angles | |
|---|---|---|---|
| R(1,5) | 1.327 | D(7,1,5,14) | 0.2383 |
| R(1,7) | 1.3271 | D(21,1,5,14) | 177.977 |
| R(1,21) | 1.4498 | D(5,1,7,13) | -0.257 |
| R(2,6) | 1.3271 | D(21,1,7,13) | -177.971 |
| R(2,12) | 1.327 | D(5,1,21,2) | -76.5418 |
| R(2,21) | 1.4498 | D(5,1,21,3) | 43.8948 |
| R(3,10) | 1.327 | D(5,1,21,4) | 164.3313 |
| R(3,11) | 1.3271 | D(7,1,21,2) | 100.9159 |
| R(3,21) | 1.4498 | D(7,1,21,3) | -138.6476 |
| R(4,8) | 1.327 | D(7,1,21,4) | -18.211 |
| R(4,9) | 1.3271 | D(12,2,6,15) | 0.2558 |
| R(4,21) | 1.4498 | D(21,2,6,15) | 177.9693 |
| R(5,14) | 1.2748 | D(6,2,12,16) | -0.2413 |
| R(6,15) | 1.2753 | D(21,2,12,16) | -177.9795 |
| R(7,13) | 1.2753 | D(6,2,21,1) | 18.2115 |
| R(8,20) | 1.2748 | D(6,2,21,3) | -100.9154 |
| R(9,19) | 1.2753 | D(6,2,21,4) | 138.6481 |
| R(10,18) | 1.2748 | D(12,2,21,1) | -164.3313 |
| R(11,17) | 1.2753 | D(12,2,21,3) | 76.5417 |
| R(12,16) | 1.2748 | D(12,2,21,4) | -43.8947 |
| R(13,14) | 1.3556 | D(11,3,10,18) | 0.237 |
| R(15,16) | 1.3556 | D(21,3,10,18) | 177.9756 |
| R(17,18) | 1.3556 | D(10,3,11,17) | -0.2601 |
| R(19,20) | 1.3556 | D(21,3,11,17) | -177.9739 |
| **Bond angles** | | D(10,3,21,1) | 43.8958 |
| A(5,1,7) | 112.0047 | D(10,3,21,2) | 164.3325 |
| A(5,1,21) | 123.5153 | D(10,3,21,4) | -76.5408 |
| A(7,1,21) | 124.4382 | D(11,3,21,1) | -138.6466 |
| A(6,2,12) | 112.0047 | D(11,3,21,2) | -18.21 |
| A(6,2,21) | 124.4381 | D(11,3,21,4) | 100.9168 |
| A(12,2,21) | 123.5153 | D(9,4,8,20) | -0.2394 |
| A(10,3,11) | 112.0046 | D(21,4,8,20) | -177.9787 |
| A(10,3,21) | 123.5154 | D(8,4,9,19) | 0.2552 |
| A(11,3,21) | 124.4381 | D(21,4,9,19) | 177.9698 |
| A(8,4,9) | 112.0047 | D(8,4,21,1) | 76.5411 |
| A(8,4,21) | 123.5153 | D(8,4,21,2) | -43.8954 |
| A(9,4,21) | 124.4382 | D(8,4,21,3) | -164.3319 |
| A(1,5,14) | 104.6827 | D(9,4,21,1) | -100.9173 |
| A(2,6,15) | 104.62 | D(9,4,21,2) | 138.6462 |
| A(1,7,13) | 104.6201 | D(9,4,21,3) | 18.2097 |
| A(4,8,20) | 104.6829 | D(1,5,14,13) | -0.123 |
| A(4,9,19) | 104.6199 | D(2,6,15,16) | -0.1658 |
| A(3,10,18) | 104.683 | D(1,7,13,14) | 0.1706 |
| A(3,11,17) | 104.6198 | D(4,8,20,19) | 0.1264 |
| A(2,12,16) | 104.6828 | D(4,9,19,20) | -0.1667 |
| A(7,13,14) | 109.3752 | D(3,10,18,17) | -0.118 |
| A(5,14,13) | 109.3167 | D(3,11,17,18) | 0.1765 |
| A(6,15,16) | 109.3754 | D(2,12,16,15) | 0.1288 |
| A(12,16,15) | 109.3165 | D(7,13,14,5) | -0.0314 |
| A(11,17,18) | 109.3757 | D(6,15,16,12) | 0.0244 |
| A(10,18,17) | 109.3162 | D(11,17,18,10) | -0.0386 |
| A(9,19,20) | 109.3756 | D(9,19,20,8) | 0.0266 |

| | |
|---|---|
| A(8,20,19) | 109.3163 |
| A(1,21,2) | 110.1953 |
| A(1,21,3) | 108.0328 |
| A(1,21,4) | 110.1953 |
| A(2,21,3) | 110.1952 |
| A(2,21,4) | 108.0326 |
| A(3,21,4) | 110.1952 |

Cartesian coordinates (standard orientation)

| Center Number | Atomic Number | Atomic Type | Coordinates (Angstroms) X | Y | Z |
|---|---|---|---|---|---|
| 1 | 7 | 0 | -0.852088 | -0.816837 | 0.841769 |
| 2 | 7 | 0 | 0.851685 | -0.842040 | -0.816970 |
| 3 | 7 | 0 | -0.851556 | 0.817248 | -0.841904 |
| 4 | 7 | 0 | 0.851956 | 0.841631 | 0.817113 |
| 5 | 7 | 0 | -1.927847 | -1.453719 | 0.396752 |
| 6 | 7 | 0 | 0.627521 | -2.127800 | -1.057241 |
| 7 | 7 | 0 | -0.628383 | -1.057136 | 2.127604 |
| 8 | 7 | 0 | 1.927434 | 0.396442 | 1.454350 |
| 9 | 7 | 0 | 0.628391 | 2.127508 | 1.057317 |
| 10 | 7 | 0 | -1.927071 | 1.454665 | -0.397061 |
| 11 | 7 | 0 | -0.627532 | 1.057423 | -2.127706 |
| 12 | 7 | 0 | 1.927486 | -0.397370 | -1.454023 |
| 13 | 7 | 0 | -1.572187 | -1.840168 | 2.477702 |
| 14 | 7 | 0 | -2.372506 | -2.084801 | 1.411272 |
| 15 | 7 | 0 | 1.571071 | -2.478192 | -1.840448 |
| 16 | 7 | 0 | 2.371751 | -1.412045 | -2.085134 |
| 17 | 7 | 0 | -1.570935 | 1.840864 | -2.477969 |
| 18 | 7 | 0 | -2.371249 | 2.085980 | -1.411646 |
| 19 | 7 | 0 | 1.571981 | 2.477445 | 1.840679 |
| 20 | 7 | 0 | 2.372077 | 1.410900 | 2.085542 |
| 21 | 6 | 0 | -0.000001 | 0.000000 | 0.000002 |

**M06/TZVP**

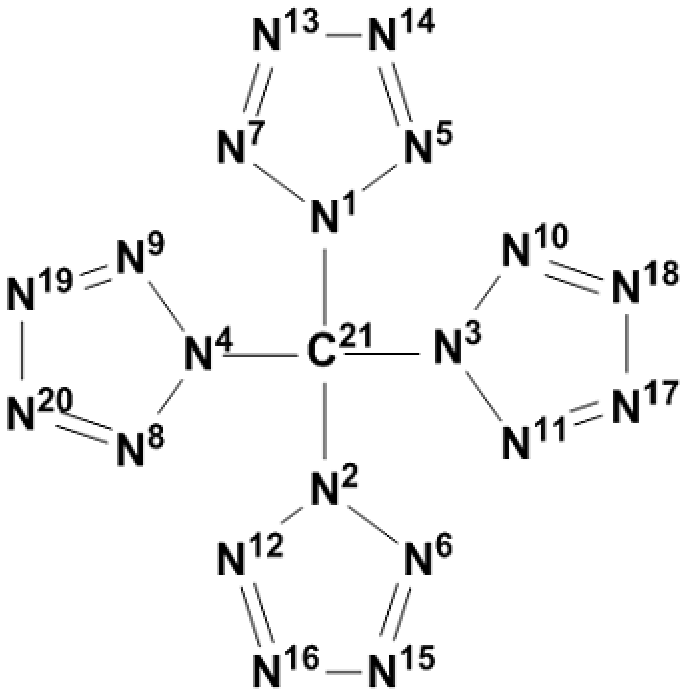


Dipole moment (Debye) = 0.0034

Mulliken charges:

| | | |
|---|---|---|
| 1 | N | 0.207697 |
| 2 | N | 0.207543 |
| 3 | N | 0.207523 |
| 4 | N | 0.207535 |
| 5 | N | -0.058726 |
| 6 | N | -0.053018 |
| 7 | N | -0.053325 |
| 8 | N | -0.058577 |
| 9 | N | -0.053395 |
| 10 | N | -0.058668 |
| 11 | N | -0.052884 |
| 12 | N | -0.058660 |
| 13 | N | -0.081426 |
| 14 | N | -0.074303 |
| 15 | N | -0.081308 |
| 16 | N | -0.074499 |
| 17 | N | -0.081466 |
| 18 | N | -0.074240 |
| 19 | N | -0.081326 |
| 20 | N | -0.074575 |
| 21 | C | 0.240097 |

Sum of Mulliken charges = -0.00000

**ΔE(multipl.=1) = 0.0 кДж/моль**

**Alpha occupied eigenvalues (highest) = -10.3781661 eV**
**Alpha virtual eigenvalues (lowest) = -3.3367623 eV**

<S**2> = 0.0000

Summary of Natural Population Analysis:

| Atom | No | Natural Charge | Natural Population Core | Valence | Rydberg | Total |
|---|---|---|---|---|---|---|
| N | 1 | -0.07438 | 1.99951 | 5.04820 | 0.02666 | 7.07438 |
| N | 2 | -0.07439 | 1.99951 | 5.04821 | 0.02667 | 7.07439 |
| N | 3 | -0.07437 | 1.99951 | 5.04819 | 0.02667 | 7.07437 |
| N | 4 | -0.07437 | 1.99951 | 5.04819 | 0.02666 | 7.07437 |
| N | 5 | 0.00136 | 1.99958 | 4.97136 | 0.02769 | 6.99864 |
| N | 6 | -0.00403 | 1.99958 | 4.97642 | 0.02802 | 7.00403 |
| N | 7 | -0.00402 | 1.99958 | 4.97641 | 0.02803 | 7.00402 |
| N | 8 | 0.00141 | 1.99958 | 4.97132 | 0.02769 | 6.99859 |
| N | 9 | -0.00406 | 1.99958 | 4.97645 | 0.02803 | 7.00406 |
| N | 10 | 0.00137 | 1.99958 | 4.97136 | 0.02769 | 6.99863 |
| N | 11 | -0.00406 | 1.99958 | 4.97645 | 0.02803 | 7.00406 |
| N | 12 | 0.00134 | 1.99958 | 4.97139 | 0.02770 | 6.99866 |
| N | 13 | -0.03483 | 1.99970 | 5.00626 | 0.02887 | 7.03483 |
| N | 14 | -0.03164 | 1.99970 | 5.00303 | 0.02891 | 7.03164 |
| N | 15 | -0.03481 | 1.99970 | 5.00623 | 0.02887 | 7.03481 |
| N | 16 | -0.03162 | 1.99970 | 5.00300 | 0.02891 | 7.03162 |
| N | 17 | -0.03480 | 1.99970 | 5.00623 | 0.02887 | 7.03480 |
| N | 18 | -0.03161 | 1.99970 | 5.00300 | 0.02891 | 7.03161 |
| N | 19 | -0.03482 | 1.99970 | 5.00625 | 0.02887 | 7.03482 |
| N | 20 | -0.03163 | 1.99970 | 5.00302 | 0.02891 | 7.03163 |
| C | 21 | 0.57395 | 1.99905 | 3.38073 | 0.04627 | 5.42605 |
| * Total * | | -0.00000 | 41.99137 | 103.40169 | 0.60694 | 146.00000 |

**Optimized Parameters**
**(Angstroms and Degrees)**

| Bond lengths | |
|---|---|
| R(1,5) | 1.3307 |
| R(1,7) | 1.3307 |
| R(1,21) | 1.444 |
| R(2,6) | 1.3307 |
| R(2,12) | 1.3307 |
| R(2,21) | 1.444 |
| R(3,10) | 1.3306 |
| R(3,11) | 1.3307 |
| R(3,21) | 1.444 |
| R(4,8) | 1.3307 |
| R(4,9) | 1.3307 |
| R(4,21) | 1.4441 |
| R(5,14) | 1.2696 |
| R(6,15) | 1.27 |
| R(7,13) | 1.27 |
| R(8,20) | 1.2696 |
| R(9,19) | 1.2699 |
| R(10,18) | 1.2696 |
| R(11,17) | 1.27 |
| R(12,16) | 1.2696 |
| R(13,14) | 1.3652 |
| R(15,16) | 1.3652 |
| R(17,18) | 1.3652 |
| R(19,20) | 1.3652 |

| Bond angles | |
|---|---|
| A(5,1,7) | 112.0221 |
| A(5,1,21) | 123.1315 |
| A(7,1,21) | 124.8278 |
| A(6,2,12) | 112.0227 |
| A(6,2,21) | 124.8297 |
| A(12,2,21) | 123.1274 |
| A(10,3,11) | 112.0221 |
| A(10,3,21) | 123.1245 |
| A(11,3,21) | 124.8342 |
| A(8,4,9) | 112.0202 |
| A(8,4,21) | 123.1377 |
| A(9,4,21) | 124.8242 |
| A(1,5,14) | 104.6839 |
| A(2,6,15) | 104.593 |
| A(1,7,13) | 104.5946 |
| A(4,8,20) | 104.686 |
| A(4,9,19) | 104.5948 |
| A(3,10,18) | 104.6874 |
| A(3,11,17) | 104.5919 |
| A(2,12,16) | 104.6853 |
| A(7,13,14) | 109.4093 |
| A(5,14,13) | 109.2896 |
| A(6,15,16) | 109.4085 |
| A(12,16,15) | 109.2901 |
| A(11,17,18) | 109.4097 |
| A(10,18,17) | 109.2886 |
| A(9,19,20) | 109.4104 |

| Torsion (dihedral) angles | |
|---|---|
| D(7,1,5,14) | 0.1997 |
| D(21,1,5,14) | 178.6972 |
| D(5,1,7,13) | -0.1878 |
| D(21,1,7,13) | -178.655 |
| D(5,1,21,2) | -74.7897 |
| D(5,1,21,3) | 45.5587 |
| D(5,1,21,4) | 165.8908 |
| D(7,1,21,2) | 103.5134 |
| D(7,1,21,3) | -136.1382 |
| D(7,1,21,4) | -15.8061 |
| D(12,2,6,15) | 0.1952 |
| D(21,2,6,15) | 178.5988 |
| D(6,2,12,16) | -0.2054 |
| D(21,2,12,16) | -178.6407 |
| D(6,2,21,1) | 15.8944 |
| D(6,2,21,3) | -103.424 |
| D(6,2,21,4) | 136.2402 |
| D(12,2,21,1) | -165.8728 |
| D(12,2,21,3) | 74.8088 |
| D(12,2,21,4) | -45.527 |
| D(11,3,10,18) | 0.2008 |
| D(21,3,10,18) | 178.6742 |
| D(10,3,11,17) | -0.1876 |
| D(21,3,11,17) | -178.63 |
| D(10,3,21,1) | 45.6121 |
| D(10,3,21,2) | 165.9613 |
| D(10,3,21,4) | -74.7247 |
| D(11,3,21,1) | -136.1121 |
| D(11,3,21,2) | -15.7629 |
| D(11,3,21,4) | 103.5511 |
| D(9,4,8,20) | -0.1898 |
| D(21,4,8,20) | -178.7183 |
| D(8,4,9,19) | 0.1766 |
| D(21,4,9,19) | 178.6756 |
| D(8,4,21,1) | 74.6866 |
| D(8,4,21,2) | -45.6621 |
| D(8,4,21,3) | -166.0048 |
| D(9,4,21,1) | -103.6515 |
| D(9,4,21,2) | 135.9998 |
| D(9,4,21,3) | 15.6571 |
| D(1,5,14,13) | -0.1295 |
| D(2,6,15,16) | -0.1061 |
| D(1,7,13,14) | 0.1001 |
| D(4,8,20,19) | 0.1249 |
| D(4,9,19,20) | -0.0922 |
| D(3,10,18,17) | -0.1313 |
| D(3,11,17,18) | 0.0988 |
| D(2,12,16,15) | 0.1313 |
| D(7,13,14,5) | 0.0195 |
| D(6,15,16,12) | -0.0167 |
| D(11,17,18,10) | 0.0216 |
| D(9,19,20,8) | -0.0216 |

| | |
|---|---|
| A(8,20,19) 109.2883<br>A(1,21,2) 110.0418<br>A(1,21,3) 108.3437<br>A(1,21,4) 110.0371<br>A(2,21,3) 110.0406<br>A(2,21,4) 108.3463<br>A(3,21,4) 110.0295 | |

Cartesian coordinates (standard orientation)

| Center Number | Atomic Number | Atomic Type | Coordinates (Angstroms) X | Y | Z |
|---|---|---|---|---|---|
| 1 | 7 | 0 | -0.738721 | 0.390927 | 1.177845 |
| 2 | 7 | 0 | 0.816301 | -1.155569 | 0.289241 |
| 3 | 7 | 0 | -0.944643 | -0.310723 | -1.046569 |
| 4 | 7 | 0 | 0.867371 | 1.075612 | -0.419372 |
| 5 | 7 | 0 | -1.798458 | -0.277136 | 1.626657 |
| 6 | 7 | 0 | 0.665348 | -1.947115 | 1.348208 |
| 7 | 7 | 0 | -0.434484 | 1.425728 | 1.957147 |
| 8 | 7 | 0 | 1.993282 | 1.392053 | 0.215392 |
| 9 | 7 | 0 | 0.632493 | 1.882667 | -1.451023 |
| 10 | 7 | 0 | -1.992209 | 0.458359 | -1.332432 |
| 11 | 7 | 0 | -0.863473 | -1.364178 | -1.855579 |
| 12 | 7 | 0 | 1.797699 | -1.570386 | -0.507925 |
| 13 | 7 | 0 | -1.305845 | 1.397957 | 2.880617 |
| 14 | 7 | 0 | -2.150037 | 0.344665 | 2.676300 |
| 15 | 7 | 0 | 1.553157 | -2.844095 | 1.206380 |
| 16 | 7 | 0 | 2.254039 | -2.611156 | 0.058192 |
| 17 | 7 | 0 | -1.859608 | -1.246870 | -2.634559 |
| 18 | 7 | 0 | -2.558319 | -0.119503 | -2.310965 |
| 19 | 7 | 0 | 1.611879 | 2.691097 | -1.454342 |
| 20 | 7 | 0 | 2.454149 | 2.387673 | -0.423625 |
| 21 | 6 | 0 | 0.000091 | -0.000007 | 0.000329 |

**M06/Def2TZVP**

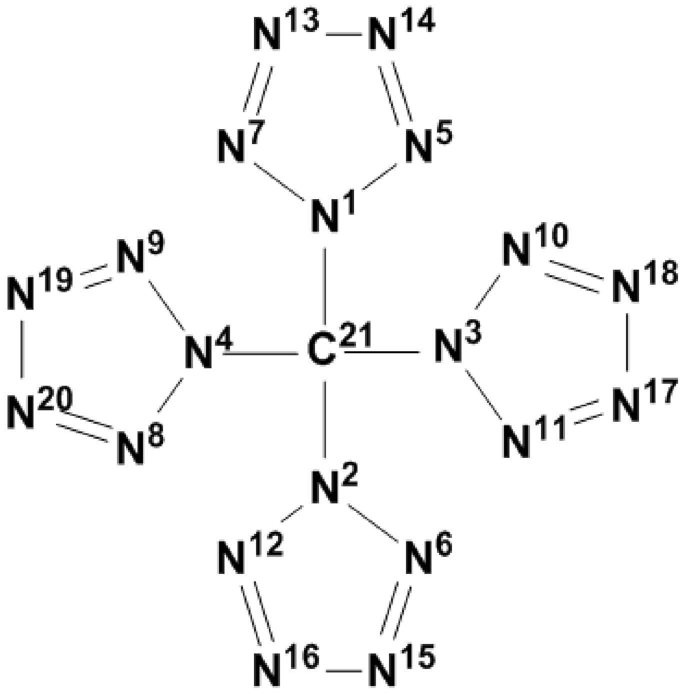


Dipole moment (Debye) = 0.0034

Mulliken charges:

| | | |
|---|---|---|
| 1 | N | 0.207697 |
| 2 | N | 0.207543 |
| 3 | N | 0.207523 |
| 4 | N | 0.207535 |
| 5 | N | -0.058726 |
| 6 | N | -0.053018 |
| 7 | N | -0.053325 |
| 8 | N | -0.058577 |
| 9 | N | -0.053395 |
| 10 | N | -0.058668 |
| 11 | N | -0.052884 |
| 12 | N | -0.058660 |
| 13 | N | -0.081426 |
| 14 | N | -0.074303 |
| 15 | N | -0.081308 |
| 16 | N | -0.074499 |
| 17 | N | -0.081466 |
| 18 | N | -0.074240 |
| 19 | N | -0.081326 |
| 20 | N | -0.074575 |
| 21 | C | 0.240097 |

Sum of Mulliken charges = -0.00000

**$\Delta$E(multipl.=1) = 0.0 кДж/моль**

**Alpha occupied eigenvalues (highest) = -10.345242 eV**
**Alpha virtual eigenvalues (lowest) = -3.2437041 eV**

<S**2> = 0.0000

Summary of Natural Population Analysis:

| Atom | No | Natural Charge | Natural Population Core | Valence | Rydberg | Total |
|---|---|---|---|---|---|---|
| N | 1 | -0.07052 | 1.99949 | 5.04094 | 0.03009 | 7.07052 |
| N | 2 | -0.07047 | 1.99949 | 5.04089 | 0.03009 | 7.07047 |
| N | 3 | -0.07050 | 1.99949 | 5.04093 | 0.03009 | 7.07050 |
| N | 4 | -0.07047 | 1.99949 | 5.04089 | 0.03009 | 7.07047 |
| N | 5 | 0.00101 | 1.99957 | 4.96530 | 0.03412 | 6.99899 |
| N | 6 | -0.00697 | 1.99957 | 4.97305 | 0.03435 | 7.00697 |
| N | 7 | -0.00697 | 1.99957 | 4.97305 | 0.03435 | 7.00697 |
| N | 8 | 0.00101 | 1.99957 | 4.96530 | 0.03413 | 6.99899 |
| N | 9 | -0.00692 | 1.99957 | 4.97301 | 0.03434 | 7.00692 |
| N | 10 | 0.00102 | 1.99957 | 4.96529 | 0.03412 | 6.99898 |
| N | 11 | -0.00693 | 1.99957 | 4.97302 | 0.03434 | 7.00693 |
| N | 12 | 0.00102 | 1.99957 | 4.96530 | 0.03412 | 6.99898 |
| N | 13 | -0.03643 | 1.99971 | 5.00108 | 0.03564 | 7.03643 |
| N | 14 | -0.03363 | 1.99971 | 4.99800 | 0.03592 | 7.03363 |
| N | 15 | -0.03643 | 1.99971 | 5.00108 | 0.03564 | 7.03643 |
| N | 16 | -0.03364 | 1.99971 | 4.99801 | 0.03593 | 7.03364 |
| N | 17 | -0.03645 | 1.99971 | 5.00110 | 0.03564 | 7.03645 |
| N | 18 | -0.03363 | 1.99971 | 4.99800 | 0.03592 | 7.03363 |
| N | 19 | -0.03644 | 1.99971 | 5.00108 | 0.03565 | 7.03644 |
| N | 20 | -0.03366 | 1.99971 | 4.99803 | 0.03593 | 7.03366 |
| C | 21 | 0.58600 | 1.99903 | 3.37676 | 0.03820 | 5.41400 |
| * Total * | | 0.00000 | 41.99118 | 103.29012 | 0.71870 | 146.00000 |

**Optimized Parameters**
**(Angstroms and Degrees)**

**Bond lengths**

| | |
|---|---|
| R(1,5) | 1.3276 |
| R(1,7) | 1.3273 |
| R(1,21) | 1.4452 |
| R(2,6) | 1.3273 |
| R(2,12) | 1.3276 |
| R(2,21) | 1.4452 |
| R(3,10) | 1.3276 |
| R(3,11) | 1.3273 |
| R(3,21) | 1.4452 |
| R(4,8) | 1.3276 |
| R(4,9) | 1.3273 |
| R(4,21) | 1.4452 |
| R(5,14) | 1.2694 |
| R(6,15) | 1.27 |
| R(7,13) | 1.27 |
| R(8,20) | 1.2694 |
| R(9,19) | 1.27 |
| R(10,18) | 1.2694 |
| R(11,17) | 1.2699 |
| R(12,16) | 1.2694 |
| R(13,14) | 1.3618 |
| R(15,16) | 1.3617 |
| R(17,18) | 1.3618 |
| R(19,20) | 1.3617 |

**Bond angles**

| | |
|---|---|
| A(5,1,7) | 111.8946 |
| A(5,1,21) | 123.4884 |
| A(7,1,21) | 124.5063 |
| A(6,2,12) | 111.8904 |
| A(6,2,21) | 124.506 |
| A(12,2,21) | 123.4965 |
| A(10,3,11) | 111.8923 |
| A(10,3,21) | 123.4824 |
| A(11,3,21) | 124.5194 |
| A(8,4,9) | 111.8914 |
| A(8,4,21) | 123.4786 |
| A(9,4,21) | 124.5203 |
| A(1,5,14) | 104.8104 |
| A(2,6,15) | 104.7627 |
| A(1,7,13) | 104.7595 |
| A(4,8,20) | 104.8129 |
| A(4,9,19) | 104.7597 |
| A(3,10,18) | 104.8111 |
| A(3,11,17) | 104.7611 |
| A(2,12,16) | 104.8136 |
| A(7,13,14) | 109.293 |
| A(5,14,13) | 109.24 |
| A(6,15,16) | 109.2925 |
| A(12,16,15) | 109.2384 |
| A(11,17,18) | 109.2929 |
| A(10,18,17) | 109.2401 |
| A(9,19,20) | 109.2925 |

**Torsion (dihedral) angles**

| | |
|---|---|
| D(7,1,5,14) | 0.4961 |
| D(21,1,5,14) | 176.8221 |
| D(5,1,7,13) | -0.5158 |
| D(21,1,7,13) | -176.7974 |
| D(5,1,21,2) | -76.3311 |
| D(5,1,21,3) | 44.059 |
| D(5,1,21,4) | 164.4585 |
| D(7,1,21,2) | 99.5313 |
| D(7,1,21,3) | -140.0786 |
| D(7,1,21,4) | -19.6791 |
| D(12,2,6,15) | 0.5121 |
| D(21,2,6,15) | 176.8545 |
| D(6,2,12,16) | -0.4943 |
| D(21,2,12,16) | -176.88 |
| D(6,2,21,1) | 19.6373 |
| D(6,2,21,3) | -99.5682 |
| D(6,2,21,4) | 140.0136 |
| D(12,2,21,1) | -164.4332 |
| D(12,2,21,3) | 76.3614 |
| D(12,2,21,4) | -44.0568 |
| D(11,3,10,18) | 0.4917 |
| D(21,3,10,18) | 176.8987 |
| D(10,3,11,17) | -0.5084 |
| D(21,3,11,17) | -176.8712 |
| D(10,3,21,1) | 44.0913 |
| D(10,3,21,2) | 164.4717 |
| D(10,3,21,4) | -76.2915 |
| D(11,3,21,1) | -139.9558 |
| D(11,3,21,2) | -19.5753 |
| D(11,3,21,4) | 99.6615 |
| D(9,4,8,20) | -0.4704 |
| D(21,4,8,20) | -176.8133 |
| D(8,4,9,19) | 0.4921 |
| D(21,4,9,19) | 176.7898 |
| D(8,4,21,1) | 76.3372 |
| D(8,4,21,2) | -44.0407 |
| D(8,4,21,3) | -164.4534 |
| D(9,4,21,1) | -99.5435 |
| D(9,4,21,2) | 140.0787 |
| D(9,4,21,3) | 19.6659 |
| D(1,5,14,13) | -0.2751 |
| D(2,6,15,16) | -0.3211 |
| D(1,7,13,14) | 0.3252 |
| D(4,8,20,19) | 0.258 |
| D(4,9,19,20) | -0.313 |
| D(3,10,18,17) | -0.2753 |
| D(3,11,17,18) | 0.3179 |
| D(2,12,16,15) | 0.2758 |
| D(7,13,14,5) | -0.0331 |
| D(6,15,16,12) | 0.0299 |
| D(11,17,18,10) | -0.0281 |
| D(9,19,20,8) | 0.0364 |

| | |
|---|---|
| A(8,20,19) 109.2412<br>A(1,21,2) 110.1114<br>A(1,21,3) 108.1734<br>A(1,21,4) 110.1089<br>A(2,21,3) 110.1269<br>A(2,21,4) 108.1864<br>A(3,21,4) 110.1357 | |

Cartesian coordinates (standard orientation)

| Center Number | Atomic Number | Atomic Type | Coordinates (Angstroms) X | Y | Z |
|---|---|---|---|---|---|
| 1 | 7 | 0 | -0.847795 | -0.807515 | 0.846306 |
| 2 | 7 | 0 | 0.847464 | -0.846575 | -0.808449 |
| 3 | 7 | 0 | -0.847371 | 0.809015 | -0.846923 |
| 4 | 7 | 0 | 0.847964 | 0.846708 | 0.808360 |
| 5 | 7 | 0 | -1.922051 | -1.451568 | 0.406129 |
| 6 | 7 | 0 | 0.609013 | -2.127755 | -1.060153 |
| 7 | 7 | 0 | -0.609554 | -1.060166 | 2.127372 |
| 8 | 7 | 0 | 1.921810 | 0.405893 | 1.452644 |
| 9 | 7 | 0 | 0.610384 | 2.127902 | 1.061145 |
| 10 | 7 | 0 | -1.920956 | 1.454050 | -0.406563 |
| 11 | 7 | 0 | -0.610393 | 1.059807 | -2.128605 |
| 12 | 7 | 0 | 1.922028 | -0.407234 | -1.452583 |
| 13 | 7 | 0 | -1.540127 | -1.849138 | 2.480047 |
| 14 | 7 | 0 | -2.352373 | -2.091730 | 1.414303 |
| 15 | 7 | 0 | 1.539669 | -2.481310 | -1.848650 |
| 16 | 7 | 0 | 2.352266 | -1.416014 | -2.091848 |
| 17 | 7 | 0 | -1.540955 | 1.848701 | -2.481383 |
| 18 | 7 | 0 | -2.352049 | 2.093011 | -1.415170 |
| 19 | 7 | 0 | 1.540686 | 2.479810 | 1.850813 |
| 20 | 7 | 0 | 2.352212 | 1.413580 | 2.093475 |
| 21 | 6 | 0 | 0.000125 | 0.000523 | -0.000264 |

**MP2/TZVP**

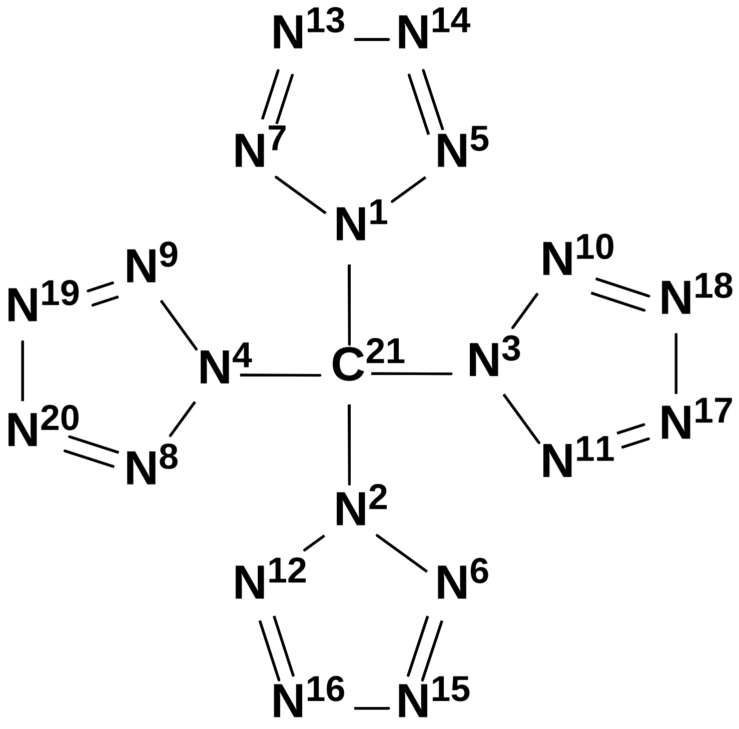


Mulliken charges:

| | | |
|---|---|---|
| 1 | N | 0.045684 |
| 2 | N | 0.045702 |
| 3 | N | 0.045706 |
| 4 | N | 0.045695 |
| 5 | N | 0.066639 |
| 6 | N | 0.040604 |
| 7 | N | 0.040608 |
| 8 | N | 0.066628 |
| 9 | N | 0.040604 |
| 10 | N | 0.066629 |
| 11 | N | 0.040598 |
| 12 | N | 0.066627 |
| 13 | N | -0.055424 |
| 14 | N | -0.045614 |
| 15 | N | -0.055418 |
| 16 | N | -0.045626 |
| 17 | N | -0.055405 |
| 18 | N | -0.045627 |
| 19 | N | -0.055419 |
| 20 | N | -0.045617 |
| 21 | C | -0.207573 |

Sum of Mulliken charges = 0.00000

Dipole moment (Debye) = 0.0001

ΔE(multipl.=1) = 0.0 кДж/моль

Alpha occupied eigenvalues (highest) = -13.9535601 eV
Alpha virtual eigenvalues (lowest) = 0.4021638 eV

<S**2> = 0.0000
Summary of Natural Population Analysis:

| Atom | No | Natural Charge | Natural Population Core | Valence | Rydberg | Total |
|---|---|---|---|---|---|---|
| N | 1 | -0.12269 | 1.99952 | 5.09462 | 0.02856 | 7.12269 |
| N | 2 | -0.12268 | 1.99952 | 5.09461 | 0.02856 | 7.12268 |
| N | 3 | -0.12269 | 1.99952 | 5.09461 | 0.02856 | 7.12269 |
| N | 4 | -0.12269 | 1.99952 | 5.09461 | 0.02856 | 7.12269 |
| N | 5 | 0.01645 | 1.99963 | 4.95743 | 0.02649 | 6.98355 |
| N | 6 | 0.01613 | 1.99963 | 4.95776 | 0.02648 | 6.98387 |
| N | 7 | 0.01613 | 1.99963 | 4.95775 | 0.02648 | 6.98387 |
| N | 8 | 0.01644 | 1.99963 | 4.95743 | 0.02649 | 6.98356 |
| N | 9 | 0.01613 | 1.99963 | 4.95776 | 0.02648 | 6.98387 |
| N | 10 | 0.01644 | 1.99963 | 4.95743 | 0.02649 | 6.98356 |
| N | 11 | 0.01613 | 1.99963 | 4.95776 | 0.02648 | 6.98387 |
| N | 12 | 0.01644 | 1.99963 | 4.95744 | 0.02649 | 6.98356 |
| N | 13 | -0.04266 | 1.99973 | 5.01265 | 0.03028 | 7.04266 |
| N | 14 | -0.03760 | 1.99973 | 5.00776 | 0.03011 | 7.03760 |
| N | 15 | -0.04266 | 1.99973 | 5.01265 | 0.03028 | 7.04266 |
| N | 16 | -0.03761 | 1.99973 | 5.00777 | 0.03011 | 7.03761 |
| N | 17 | -0.04265 | 1.99973 | 5.01264 | 0.03028 | 7.04265 |
| N | 18 | -0.03761 | 1.99973 | 5.00777 | 0.03011 | 7.03761 |
| N | 19 | -0.04265 | 1.99973 | 5.01264 | 0.03028 | 7.04265 |
| N | 20 | -0.03761 | 1.99973 | 5.00777 | 0.03011 | 7.03761 |
| C | 21 | 0.68151 | 1.99917 | 3.28127 | 0.03806 | 5.31849 |
| * Total * | | 0.00000 | 41.99215 | 103.40212 | 0.60573 | 146.00000 |

**Optimized Parameters**
**(Angstroms and Degrees)**

| Bond lengths | |
|---|---|
| R(1,5) | 1.3338 |
| R(1,7) | 1.3354 |
| R(1,21) | 1.4413 |
| R(2,6) | 1.3354 |
| R(2,12) | 1.3338 |
| R(2,21) | 1.4413 |
| R(3,10) | 1.3338 |
| R(3,11) | 1.3354 |
| R(3,21) | 1.4413 |
| R(4,8) | 1.3338 |
| R(4,9) | 1.3354 |
| R(4,21) | 1.4413 |
| R(5,14) | 1.3121 |
| R(6,15) | 1.3123 |
| R(7,13) | 1.3123 |
| R(8,20) | 1.3121 |
| R(9,19) | 1.3123 |
| R(10,18) | 1.3121 |
| R(11,17) | 1.3123 |
| R(12,16) | 1.3121 |
| R(13,14) | 1.3526 |
| R(15,16) | 1.3526 |
| R(17,18) | 1.3526 |
| R(19,20) | 1.3526 |

| Bond angles | |
|---|---|
| A(5,1,7) | 113.8864 |
| A(5,1,21) | 121.762 |
| A(7,1,21) | 124.349 |
| A(6,2,12) | 113.8871 |
| A(6,2,21) | 124.3485 |
| A(12,2,21) | 121.7618 |
| A(10,3,11) | 113.8858 |
| A(10,3,21) | 121.7621 |
| A(11,3,21) | 124.3494 |
| A(8,4,9) | 113.8864 |
| A(8,4,21) | 121.7621 |
| A(9,4,21) | 124.3489 |
| A(1,5,14) | 103.4613 |
| A(2,6,15) | 103.2609 |
| A(1,7,13) | 103.2605 |
| A(4,8,20) | 103.4599 |
| A(4,9,19) | 103.262 |
| A(3,10,18) | 103.4596 |
| A(3,11,17) | 103.2628 |
| A(2,12,16) | 103.46 |
| A(7,13,14) | 109.8156 |
| A(5,14,13) | 109.5758 |
| A(6,15,16) | 109.8146 |
| A(12,16,15) | 109.5771 |
| A(11,17,18) | 109.8123 |
| A(10,18,17) | 109.5791 |
| A(9,19,20) | 109.8134 |

| Torsion (dihedral) angles | |
|---|---|
| D(7,1,5,14) | -0.1912 |
| D(21,1,5,14) | -179.626 |
| D(5,1,7,13) | 0.1893 |
| D(21,1,7,13) | 179.6071 |
| D(5,1,21,2) | -73.8366 |
| D(5,1,21,3) | 46.5513 |
| D(5,1,21,4) | 166.9391 |
| D(7,1,21,2) | 106.7894 |
| D(7,1,21,3) | -132.8227 |
| D(7,1,21,4) | -12.4348 |
| D(12,2,6,15) | -0.1939 |
| D(21,2,6,15) | -179.6144 |
| D(6,2,12,16) | 0.1884 |
| D(21,2,12,16) | 179.6256 |
| D(6,2,21,1) | 12.4421 |
| D(6,2,21,3) | -106.7826 |
| D(6,2,21,4) | 132.83 |
| D(12,2,21,1) | -166.9347 |
| D(12,2,21,3) | 73.8407 |
| D(12,2,21,4) | -46.5467 |
| D(11,3,10,18) | -0.1868 |
| D(21,3,10,18) | -179.6228 |
| D(10,3,11,17) | 0.1963 |
| D(21,3,11,17) | 179.6155 |
| D(10,3,21,1) | 46.553 |
| D(10,3,21,2) | 166.9406 |
| D(10,3,21,4) | -73.8352 |
| D(11,3,21,1) | -132.8224 |
| D(11,3,21,2) | -12.4348 |
| D(11,3,21,4) | 106.7894 |
| D(9,4,8,20) | 0.1882 |
| D(21,4,8,20) | 179.6201 |
| D(8,4,9,19) | -0.1957 |
| D(21,4,9,19) | -179.6107 |
| D(8,4,21,1) | 73.8351 |
| D(8,4,21,2) | -46.5525 |
| D(8,4,21,3) | -166.9401 |
| D(9,4,21,1) | -106.7941 |
| D(9,4,21,2) | 132.8184 |
| D(9,4,21,3) | 12.4307 |
| D(1,5,14,13) | 0.1124 |
| D(2,6,15,16) | 0.1183 |
| D(1,7,13,14) | -0.108 |
| D(4,8,20,19) | -0.1011 |
| D(4,9,19,20) | 0.1213 |
| D(3,10,18,17) | 0.0984 |
| D(3,11,17,18) | -0.1236 |
| D(2,12,16,15) | -0.1032 |
| D(7,13,14,5) | -0.0027 |
| D(6,15,16,12) | -0.0101 |
| D(11,17,18,10) | 0.0168 |
| D(9,19,20,8) | -0.0135 |

| | |
|---|---|
| A(8,20,19) 109.578<br>A(1,21,2) 110.1129<br>A(1,21,3) 108.1949<br>A(1,21,4) 110.1136<br>A(2,21,3) 110.1133<br>A(2,21,4) 108.1945<br>A(3,21,4) 110.113 | |

Cartesian coordinates (standard orientation)

| Center Number | Atomic Number | Atomic Type | Coordinates (Angstroms) X | Y | Z |
|---|---|---|---|---|---|
| 1 | 7 | 0 | 0.850886 | 0.675977 | -0.946741 |
| 2 | 7 | 0 | -0.840168 | -0.952732 | -0.680897 |
| 3 | 7 | 0 | 0.839411 | -0.674868 | 0.957658 |
| 4 | 7 | 0 | -0.850126 | 0.951690 | 0.669922 |
| 5 | 7 | 0 | 1.898367 | 0.069544 | -1.507143 |
| 6 | 7 | 0 | -0.666437 | -1.341114 | -1.946656 |
| 7 | 7 | 0 | 0.687407 | 1.942772 | -1.336205 |
| 8 | 7 | 0 | -1.889964 | 1.518216 | 0.056078 |
| 9 | 7 | 0 | -0.693436 | 1.340122 | 1.937891 |
| 10 | 7 | 0 | 1.878755 | -0.067107 | 1.531598 |
| 11 | 7 | 0 | 0.672517 | -1.941877 | 1.344986 |
| 12 | 7 | 0 | -1.887207 | -1.520498 | -0.080599 |
| 13 | 7 | 0 | 1.675943 | 2.128511 | -2.179103 |
| 14 | 7 | 0 | 2.408696 | 0.996328 | -2.283122 |
| 15 | 7 | 0 | -1.647790 | -2.191097 | -2.138122 |
| 16 | 7 | 0 | -2.386557 | -2.300096 | -1.010333 |
| 17 | 7 | 0 | 1.650163 | -2.126354 | 2.200762 |
| 18 | 7 | 0 | 2.380185 | -0.993305 | 2.314058 |
| 19 | 7 | 0 | -1.678254 | 2.188863 | 2.116636 |
| 20 | 7 | 0 | -2.402393 | 2.297042 | 0.979315 |
| 21 | 6 | 0 | 0.000003 | 0.000018 | -0.000014 |

**MP3/TZVP**

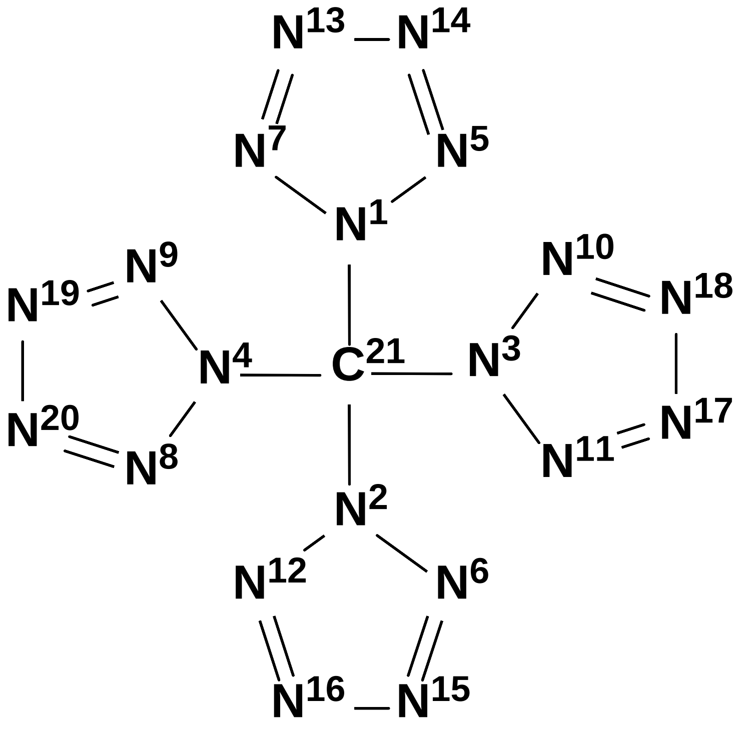


Mulliken charges:

| | | |
|---|---|---|
| 1 | N | 0.032072 |
| 2 | N | 0.032479 |
| 3 | N | 0.032403 |
| 4 | N | 0.032025 |
| 5 | N | 0.074343 |
| 6 | N | 0.032002 |
| 7 | N | 0.031978 |
| 8 | N | 0.074255 |
| 9 | N | 0.031895 |
| 10 | N | 0.074403 |
| 11 | N | 0.031942 |
| 12 | N | 0.074473 |
| 13 | N | -0.045607 |
| 14 | N | -0.044102 |
| 15 | N | -0.045541 |
| 16 | N | -0.043949 |
| 17 | N | -0.045655 |
| 18 | N | -0.043948 |
| 19 | N | -0.045726 |
| 20 | N | -0.044099 |
| 21 | C | -0.195643 |

Sum of Mulliken charges = -
0.00000

Dipole moment (Debye) = 0.0078

ΔE(multipl.=1) = 0.0 кДж/моль

Alpha occupied eigenvalues (highest) = -14.1252552 eV
Alpha virtual eigenvalues (lowest) = 0.7841922 eV

<S**2> = 0.0000

Summary of Natural Population Analysis:

| Atom | No | Natural Charge | Natural Population Core | Valence | Rydberg | Total |
|---|---|---|---|---|---|---|
| N | 1 | -0.13594 | 1.99952 | 5.10690 | 0.02951 | 7.13594 |
| N | 2 | -0.13591 | 1.99952 | 5.10687 | 0.02951 | 7.13591 |
| N | 3 | -0.13594 | 1.99952 | 5.10690 | 0.02951 | 7.13594 |
| N | 4 | -0.13593 | 1.99952 | 5.10690 | 0.02951 | 7.13593 |
| N | 5 | 0.02767 | 1.99961 | 4.94475 | 0.02798 | 6.97233 |
| N | 6 | 0.02151 | 1.99961 | 4.95092 | 0.02796 | 6.97849 |
| N | 7 | 0.02154 | 1.99961 | 4.95090 | 0.02796 | 6.97846 |
| N | 8 | 0.02767 | 1.99961 | 4.94475 | 0.02798 | 6.97233 |
| N | 9 | 0.02153 | 1.99961 | 4.95090 | 0.02796 | 6.97847 |
| N | 10 | 0.02767 | 1.99961 | 4.94475 | 0.02798 | 6.97233 |
| N | 11 | 0.02153 | 1.99961 | 4.95090 | 0.02796 | 6.97847 |
| N | 12 | 0.02765 | 1.99961 | 4.94477 | 0.02798 | 6.97235 |
| N | 13 | -0.04396 | 1.99971 | 5.01282 | 0.03143 | 7.04396 |
| N | 14 | -0.04095 | 1.99971 | 5.00993 | 0.03131 | 7.04095 |
| N | 15 | -0.04397 | 1.99971 | 5.01282 | 0.03143 | 7.04397 |
| N | 16 | -0.04094 | 1.99971 | 5.00992 | 0.03131 | 7.04094 |
| N | 17 | -0.04397 | 1.99971 | 5.01283 | 0.03143 | 7.04397 |
| N | 18 | -0.04093 | 1.99971 | 5.00992 | 0.03131 | 7.04093 |
| N | 19 | -0.04397 | 1.99971 | 5.01283 | 0.03143 | 7.04397 |
| N | 20 | -0.04094 | 1.99971 | 5.00992 | 0.03131 | 7.04094 |
| C | 21 | 0.68658 | 1.99920 | 3.27647 | 0.03775 | 5.31342 |
| * Total * | | -0.00000 | 41.99181 | 103.37767 | 0.63052 | 146.00000 |

**Optimized Parameters**
**(Angstroms and Degrees)**

| Bond lengths | | Torsion (dihedral) angles | |
|---|---|---|---|
| R(1,5) | 1.3283 | D(7,1,5,14) | 0.3804 |
| R(1,7) | 1.3284 | D(21,1,5,14) | 178.1225 |
| R(1,21) | 1.4439 | D(5,1,7,13) | -0.3821 |
| R(2,6) | 1.3284 | D(21,1,7,13) | -178.0892 |
| R(2,12) | 1.3283 | D(5,1,21,2) | -75.8931 |
| R(2,21) | 1.4439 | D(5,1,21,3) | 44.5213 |
| R(3,10) | 1.3283 | D(5,1,21,4) | 164.9376 |
| R(3,11) | 1.3284 | D(7,1,21,2) | 101.5757 |
| R(3,21) | 1.4439 | D(7,1,21,3) | -138.0099 |
| R(4,8) | 1.3283 | D(7,1,21,4) | -17.5936 |
| R(4,9) | 1.3284 | D(12,2,6,15) | 0.3808 |
| R(4,21) | 1.4439 | D(21,2,6,15) | 178.0843 |
| R(5,14) | 1.275 | D(6,2,12,16) | -0.3778 |
| R(6,15) | 1.2755 | D(21,2,12,16) | -178.1161 |
| R(7,13) | 1.2755 | D(6,2,21,1) | 17.5943 |
| R(8,20) | 1.2751 | D(6,2,21,3) | -101.5746 |
| R(9,19) | 1.2755 | D(6,2,21,4) | 138.0104 |
| R(10,18) | 1.2751 | D(12,2,21,1) | -164.9411 |
| R(11,17) | 1.2755 | D(12,2,21,3) | 75.8901 |
| R(12,16) | 1.2751 | D(12,2,21,4) | -44.525 |
| R(13,14) | 1.3647 | D(11,3,10,18) | 0.3761 |
| R(15,16) | 1.3647 | D(21,3,10,18) | 178.1271 |
| R(17,18) | 1.3647 | D(10,3,11,17) | -0.376 |
| R(19,20) | 1.3647 | D(21,3,11,17) | -178.0923 |
| **Bond angles** | | D(10,3,21,1) | 44.5421 |
| A(5,1,7) | 112.4008 | D(10,3,21,2) | 164.9572 |
| A(5,1,21) | 123.1221 | D(10,3,21,4) | -75.8744 |
| A(7,1,21) | 124.4353 | D(11,3,21,1) | -137.9791 |
| A(6,2,12) | 112.4016 | D(11,3,21,2) | -17.564 |
| A(6,2,21) | 124.4335 | D(11,3,21,4) | 101.6045 |
| A(12,2,21) | 123.123 | D(9,4,8,20) | -0.3773 |
| A(10,3,11) | 112.4009 | D(21,4,8,20) | -178.134 |
| A(10,3,21) | 123.1231 | D(8,4,9,19) | 0.3797 |
| A(11,3,21) | 124.4347 | D(21,4,9,19) | 178.1017 |
| A(8,4,9) | 112.401 | D(8,4,21,1) | 75.8754 |
| A(8,4,21) | 123.1224 | D(8,4,21,2) | -44.5404 |
| A(9,4,21) | 124.4354 | D(8,4,21,3) | -164.9546 |
| A(1,5,14) | 104.5383 | D(9,4,21,1) | -101.6097 |
| A(2,6,15) | 104.458 | D(9,4,21,2) | 137.9745 |
| A(1,7,13) | 104.459 | D(9,4,21,3) | 17.5602 |
| A(4,8,20) | 104.5385 | D(1,5,14,13) | -0.2223 |
| A(4,9,19) | 104.4583 | D(2,6,15,16) | -0.2277 |
| A(3,10,18) | 104.5385 | D(1,7,13,14) | 0.2271 |
| A(3,11,17) | 104.458 | D(4,8,20,19) | 0.2197 |
| A(2,12,16) | 104.538 | D(4,9,19,20) | -0.2265 |
| A(7,13,14) | 109.3423 | D(3,10,18,17) | -0.2215 |
| A(5,14,13) | 109.2582 | D(3,11,17,18) | 0.2218 |
| A(6,15,16) | 109.3432 | D(2,12,16,15) | 0.2195 |
| A(12,16,15) | 109.2578 | D(7,13,14,5) | -0.0032 |
| A(11,17,18) | 109.3439 | D(6,15,16,12) | 0.0055 |
| A(10,18,17) | 109.2573 | D(11,17,18,10) | -0.0003 |
| A(9,19,20) | 109.3434 | D(9,19,20,8) | 0.0045 |

| | | |
|---|---|---|
| A(8,20,19) | 109.2574 | |
| A(1,21,2) | 110.1598 | |
| A(1,21,3) | 108.1031 | |
| A(1,21,4) | 110.1603 | |
| A(2,21,3) | 110.1587 | |
| A(2,21,4) | 108.1027 | |
| A(3,21,4) | 110.16 | |

Cartesian coordinates (standard orientation)

| Center Number | Atomic Number | Atomic Type | Coordinates (Angstroms) X | Y | Z |
|---|---|---|---|---|---|
| 1 | 7 | 0 | 0.853355 | 0.689480 | -0.938840 |
| 2 | 7 | 0 | -0.842607 | -0.944787 | -0.694374 |
| 3 | 7 | 0 | 0.841941 | -0.688323 | 0.949794 |
| 4 | 7 | 0 | -0.852676 | 0.943896 | 0.683389 |
| 5 | 7 | 0 | 1.925485 | 0.131811 | -1.490143 |
| 6 | 7 | 0 | -0.616072 | -1.390163 | -1.925241 |
| 7 | 7 | 0 | 0.637255 | 1.921673 | -1.385773 |
| 8 | 7 | 0 | -1.917384 | 1.501751 | 0.118063 |
| 9 | 7 | 0 | -0.643043 | 1.389048 | 1.917336 |
| 10 | 7 | 0 | 1.905962 | -0.129272 | 1.515240 |
| 11 | 7 | 0 | 0.621842 | -1.920993 | 1.393449 |
| 12 | 7 | 0 | -1.914020 | -1.503724 | -0.142989 |
| 13 | 7 | 0 | 1.585587 | 2.131449 | -2.212595 |
| 14 | 7 | 0 | 2.381945 | 1.025072 | -2.277211 |
| 15 | 7 | 0 | -1.557222 | -2.223771 | -2.140530 |
| 16 | 7 | 0 | -2.359558 | -2.294094 | -1.038872 |
| 17 | 7 | 0 | 1.559537 | -2.129602 | 2.232603 |
| 18 | 7 | 0 | 2.353376 | -1.022107 | 2.307970 |
| 19 | 7 | 0 | -1.587974 | 2.221415 | 2.120444 |
| 20 | 7 | 0 | -2.375738 | 2.291201 | 1.008255 |
| 21 | 6 | 0 | 0.000008 | 0.000075 | -0.000006 |
